# Clinical research of patients with multiple organ dysfunction syndrome induced by severe heat stroke: nine case reports and literature review

**DOI:** 10.1186/cc13264

**Published:** 2014-03-17

**Authors:** J Liu, G Zou, Y Wu, W Li

**Affiliations:** 1 Suzhou Municipal Hospital (Eastern) Nanjing Medical University, Suzhou, China; 2 Jinling Hospital, Nanjing University School of Medicine, Nanjing, China

## Introduction

The objective was to analyze the clinical features of multiple organ dysfunction syndrome (MODS) induced by severe heat stroke, and to investigate the pathogenesis, diagnosis and prevention strategies in patients with MODS caused by severe heat stroke.

## Methods

We retrospectively studied nine cases of MODS caused by severe heat stroke, and systemically reviewed the relevant literature.

## Results

(1) Nine patients with MODS caused by severe heat stroke were all exposed in hot and humid environments. All nine patients are severe heat stroke, including seven cases of classic heat stroke (77.8%) and two cases of exertional heat stroke (22.2%). (2) Among nine cases of MODS caused by severe heat stroke, eight patients met the diagnostic criteria for SIRS. In addition, there was a significant increase in blood PMN proportion and serum CRP of all nine patients. Of note, there was also a marked increase in serum IL-6 (average (36 ± 19) pg/ml; reference range (0 to 5.9 pg/ml)); and TNFa level (average (21 ± 10) pg/ml; reference range (0 to 8.1 pg/ml)), while IL-8 was normal (average (22 ± 25) pg/ ml; reference range (0 to 62 pg/ml)). (3) There were 34 organ damages involved in all nine patients with MODS induced by severe heat stroke. The kidney, circulatory and liver accounted for 58.8% of all these organ dysfunctions. The incidence of severe heatstroke-induced organ dysfunction in turn was the kidney, circulation, liver, blood coagulation, metabolism, brain, lungs, and gastrointestinal tract. (4) During hospitalization, the common complication was pulmonary infection in patients with MODS caused by heat stroke. (5) After early intensive care of organ supportive treatment, there was a quick improvement and recovery in most cases in several days. Seven patients survived, and the average length of stay in hospital was 9.5 days.

## Conclusion

Severe heat stroke results in significant abnormal changes in inflammatory markers in patients with MODS induced by heat stroke. The types of organ dysfunction in heat stroke-induced MODS are usually distinct from those of infection and trauma. After active cooling and intensive organ function supportive treatment, most patients recovered in a relatively short period.

